# Paraneoplastic granulocyte colony-stimulating factor secretion in soft tissue sarcoma mimicking myeloproliferative neoplasia: a case report

**DOI:** 10.1186/1756-0500-7-313

**Published:** 2014-05-23

**Authors:** Christiane Dorn, Stefanie Bugl, Elke Malenke, Martin R Müller, Katja C Weisel, Ulrich Vogel, Marius Horger, Lothar Kanz, Hans-Georg Kopp

**Affiliations:** 1 Department of Medical Oncology, Hematology, Immunology, Rheumatology and Pulmology, Medical Center II, South West German Comprehensive Cancer Center, University Hospital of Tuebingen, Tuebingen, Germany; 2 Department of Pathology, South West German Comprehensive Cancer Center, Tuebingen, Germany; 3 Department of Radiology, South West German Comprehensive Cancer Center, Eberhard-Karls-University, Tuebingen, Germany

**Keywords:** Paraneoplastic leukocytosis, Neutrophilia, G-CSF, Sarcoma

## Abstract

**Background:**

While paraneoplastic leukocytosis is a common phenomenon in solid tumors, extreme elevations of white blood counts (WBC) in the range of more than 100,000/μl are uncommon in patients with non-hematologic malignancies. Leukocytosis with mature neutrophils due to a granulocyte colony-stimulating factor (G-CSF) producing tumor is only seen on rare occasions.

**Case presentation:**

Massive neutrophil leukocytosis of approximately 100,000/μl was diagnosed in a 57-year-old Caucasian woman with metastatic undifferentiated endometrial sarcoma. A bone marrow trephine biopsy revealed massively increased granulopoiesis, but no evidence of monoclonal myeloproliferative disease. After the primary tumor had been resected, white blood count (WBC) plummeted and went back to nearly normal levels within one week. With progressive metastatic disease, granulocyte colony-stimulating factor (G-CSF) plasma levels were found to be increased by 10-fold. White blood count (WBC) strictly correlated with tumor burden and response to chemotherapy. In the final stage of treatment resistent disease, white blood count (WBC) approximated 300,000/μl.

**Conclusion:**

We report on a granulocyte colony-stimulating factor (G-CSF) secreting undifferentiated endometrial sarcoma, which was associated with extreme neutrophil counts. White blood count (WBC) were closely correlated with tumor burden and associated with an aggressive clinical course. We suggest that paraneoplastic neutrophilia represents a poor prognostic sign in soft tissue sarcoma. In patients with similar constellations, antitumor therapy must not be delayed.

## Background

Paraneoplastic leukocytosis is a common phenomenon in several different solid tumors. Affected patients typically have total white blood counts (WBC) between 12,000 and 30,000/μl with mainly mature neutrophil granulocytes [1].

Idiopathic extreme leukocytosis, also known as leukemoid reaction and defined as WBC ≥ 40,000/μl is rare in patients with non-hematologic malignancies: in the largest retrospective analysis of these uncommon patients, only 10% of cases were paraneoplastic in origin. Ninety-six percent of these patients had neutrophilia, and the mean WBC was 53,000/μl [2]. A severe left shift, i.e. the presence of immature granulocytes such as myelocytes, promyelocytes, and blasts suggests myeloproliferative disorders, while paraneoplastic neutrophilia typically presents with mature neutrophils only. Leukocytosis with non-clonally derived mature neutrophils, due to paraneoplastic production of granulocyte colony-stimulating factor (G-CSF) is a rare phenomenon and has been described in a variety of different tumors [2,3]. Most cases are associated with gastrointestinal, lung, or genitourinary carcinomas [4-8]. G-CSF producing tumors are typically poorly differentiated and patients are diagnosed in an advanced stage with a large tumor burden [9,10]. Accordingly, disease outcomes are poor, which has been postulated to represent autocrine stimulation of tumor growth by G-CSF [11,12]. In this article we report on a patient with a G-CSF producing undifferentiated endometrial sarcoma with massive neutrophil leukocytosis and an aggressive clinical course.

## Case presentation

**Figure 1:**
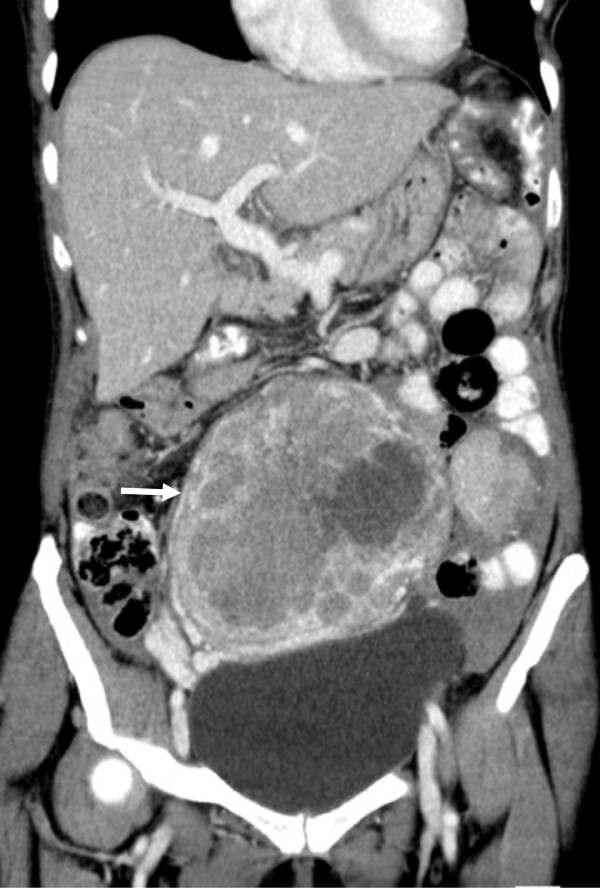
Abdominal computed tomography scan with primary tumor (14 × 10 × 12 cm) in close relation to the uterus, with peritoneal nodules and ascites (white arrow).

**Figure 2:**
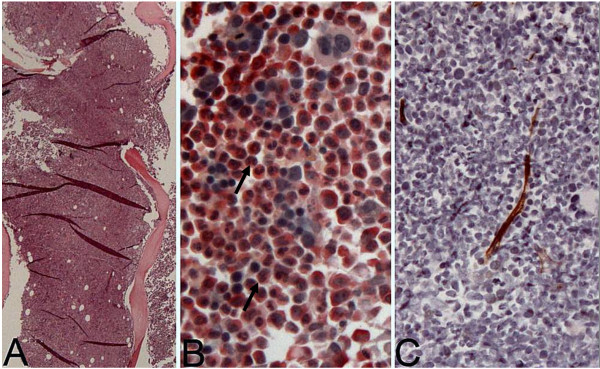
**Bone marrow trephine biopsy performed on admission. A)** Hematoxylin & eosin staining, 400x magnification. **B)** Naphthol-ASD-chloroacetate esterase stain, 400x magnification, showing marked hypercellularity due to granulocytic hyperproliferation (black arrows). **C)** Anti-CD34 immunohistochemistry reveals no detectable increase in CD34+ cells, 200x magnification.

Herein, we report on a 57-year-old Caucasian woman, who presented to our emergency department on July 30th 2011 with severe abdominal pain and a palpable lower abdominal tumor. A computed tomography (CT) scan revealed a 14 × 10 × 12 cm mass with close relation to the uterus (Figure 1). Multiple pulmonary and peritoneal nodules consistent with metastatic disease were seen. Blood counts revealed massive leukocytosis (WBC 96,910/μl) and anemia with a hemoglobin concentration of 8 g/dl. A differential hemogram revealed immature precursor cells such as promyelocytes and myelocytes as well as pronounced neutrophilia with 88% neutrophils. Lactate dehydrogenase (LDH) was slightly elevated (260 U/l), but the remaining laboratory values were within normal limits (Table 1). A bone marrow biopsy was performed at the same day. Figure 2A shows the trephine biopsy at 400x magnification stained with hematoxylin & eosin (H&E). Figure 2B reveals marked hypercellularity due to granulocytic proliferation (naphthol-ASD-chloroacetate esterase stain, 400x). Immunohistochemistry with anti-CD34 stained vascular endothelium as positive control, but there was no detecteable increase in CD34+ cells (Figure 2C, 200x). Moreover, there were no signs of myelodysplasia. Cytogenetic and molecular analyses revealed neither translocation t(9;22) nor a *BCR/ABL* fusion gene. In addition, analysis of *JAK2-V617F* mutation was performed and showed a negative result.

**Table 1:** Differential blood counts after surgery

**Parameter**	**Day 0**	**Day 0 2 h postop.**	**Day 3 postop.**	**Reference values**
**WBC (μl**^**−1**^**)**	96.910	41.410	15.050	4.000 – 9.000
**Neutrophils (%)**	88	n.d.	84.7	40 – 80
**Hemoglobin (g/dl)**	8.0	8.8	10.0	14.0 – 18.0
**Platelets (μl**^**−1**^**)**	298.000	59.000	146.000	150.000 – 450.000
**C-reactive protein (mg/dl)**	0.84	0.95	2.35	< 0.5
**LDH (U/l)**	260	n.d.	n.d.	<250

n.d.: not determined; WBC: white blood count; LDH: lactate dehydrogenase; postop.: postoperative.

**Figure 3:**
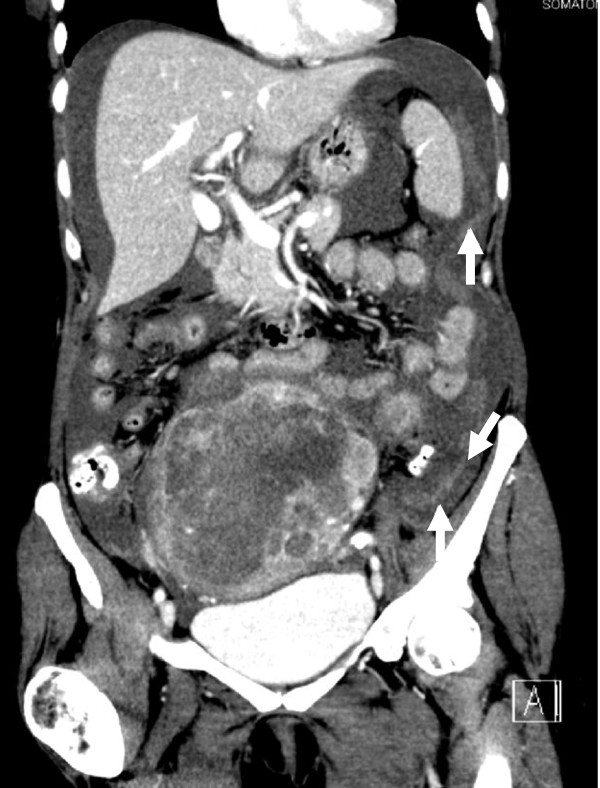
**Abdominal computed tomography scan (A) 12 hours after first computed tomography scan showing diffuse intraabdominal bleeding (white arrows).** There is increase in the free abdominal fluid and inhomogeneity of the abdominal mass.

Simultaneously, the general condition of the patient rapidly worsened over night and a further hemoglobin decline was diagnosed. Another CT scan on the following day confirmed abdominal bleeding (white arrows, Figure 3). An emergency operation including hysterectomy and bilateral adnexectomy was performed. Intraoperatively, a grossly enlarged, partly ruptured uterus with spontaneous intraperitoneal bleeding was found. A concurrently causative bleeding disturbance was excluded by routine laboratory examinations. Histology revealed undifferentiated endometrial sarcoma (FNCLCC grade 3).

**Figure 4:**
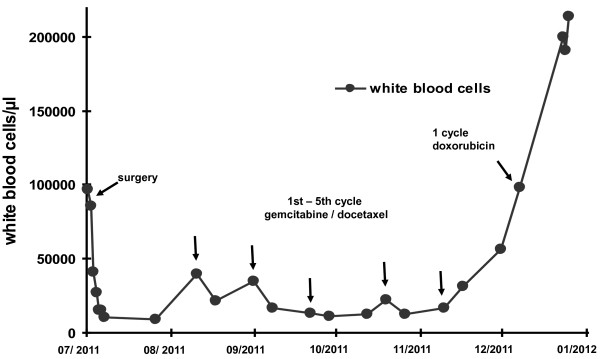
**Blood counts after surgery.** Note that white blood counts decreased immediately after surgery and strictly correlated with the further course of disease (decrease after each cycle of chemotherapy, rapid increase in correlation to fulminant disease progression).

Only few hours after tumor resection, leukocyte and platelet counts decreased rapidly. Within one week, blood counts returned to near normal levels. Figure 4 depicts the rapid decline of WBC immediately after surgery.

**Figure 5:**
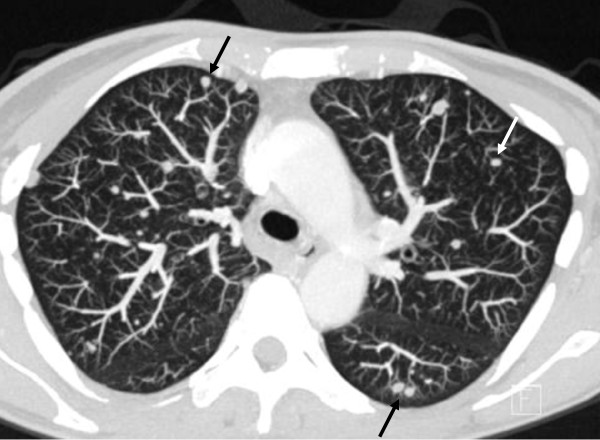
Computed tomography scan of the thorax after five cycles of chemotherapy showing multiple metastatic lesions (black and white arrows) corresponding to pulmonary metastasis.

Unfortunately, the first postoperative CT scan documented rapid progression of bilateral disseminated pulmonary metastases (Figure 5). In parallel, WBC increased, and chemotherapy with gemcitabine and docetaxel was initiated [13]. Temporary stabilization was achievable, and WBC correlated strictly with tumor burden, showing a significant increase after 5 cycles of chemotherapy. At that time, another CT scan confirmed considerable growth of all preexisting and multiple new tumor manifestations. Because paraneoplastic G-CSF secretion was suspected, G-CSF plasma levels were analyzed by enzyme linked immunosorbent assay (ELISA, RnD Systems) and found to be enormously increased to 1,270 pg/ml, corresponding to about 10 times the upper limit of normal (range 12,5 – 142 pg/ml). At the same day, WBC had reached almost 200,000/μl (Figure 4). The performance status of the patient worsened rapidly, certainly due to the massive tumor growth with organ infiltration but also due to a developing leukostasis syndrome. Second-line chemotherapy with doxorubicin was initiated, but proved to be ineffective in controlling the disease. After 2 cycles, chemotherapy was discontinued. The patient passed away within few weeks due to fulminant disease progression.

## Conclusion

In summary, this case demonstrates severe leukemoid reaction with pathologic left shift caused by a G-CSF secreting advanced undifferentiated endometrial sarcoma imitating hematologic malignancy.

Consistent with previously reported cases of paraneoplastic G-CSF secretion, leukemoid reaction was closely correlated with tumor burden and associated with an aggressive clinical course. To our knowledge, neutrophilic leukocytosis up to 200,000/μl and a severe left shift with massively increased G-CSF levels has not been reported before as a paraneoplastic phenomenon in sarcoma. We suggest not to delay antitumor treatment in patients with a similar constellation, but rather aggressively treat the underlying solid malignancy utilizing a response-inducing chemotherapy regimen.

## Consent

Written informed consent was obtained from the patient herself before blood was drawn for G-CSF analysis. The patient agreed to publication of this case report and accompanying images. A copy of the written consent is available for review by the Editor-in-Chief of this journal.

## Competing interests

The authors declare that they have no competing interests.

## Authors’ contributions

CD and HGK wrote the manuscript. SB and EM performed laboratory analyses. MRM, KCW, and LK revised and corrected the manuscript. UV has processed tumor tissue inclusive immunohistochemistry. MH provided CT-imaging. All authors read and approved the final manuscript.
